# Exploring the impact of hAMSCs secretome on Panc1 cells via TNF-α/NF-κB (p50/p65)/Caspase 3 signaling pathways: An *in vitro* study

**DOI:** 10.1016/j.bbrep.2025.102411

**Published:** 2025-12-12

**Authors:** Aynaz Khalafi, Fatemeh Safari

**Affiliations:** Department of Biology, Faculty of Science, University of Guilan, Rasht, Iran

**Keywords:** TNF-α, NF-κB, Panc1 pancreatic cancer cells, Secretome of hAMSCs, Apoptosis

## Abstract

The second most prevalent cause of mortality worldwide is cancer. Pancreatic cancer, known as the “king of cancers” due to its unfavorable prognosis and absence of symptoms, is among the fatal types of cancer. Despite the availability of various cancer therapy options, current strategies are often ineffective. Therefore, there is a constant need to explore novel platforms with low side effects and high efficacy. The application of stem cells or their derivatives in treating diseases including cancer, has become well-established. This study, focuses on investigating the effects of the secretome of human mesenchymal stem cells (hAMSCs) on Panc1 pancreatic cancer cells through the tumor necrosis factor-alpha (TNF-α)/nuclear factor-κB (NF-κB)/Caspase 3 signaling pathways. A co-culture system using 6-well plates transwell was utilized for this purpose. After 72 h, cell death in hAMSCs-treated Panc1 cells was analyzed through the TNF-α/NF-κB (p50/p65)**/**Caspase 3 signaling pathways using Western blot and enzyme-linked immunosorbent assay (ELISA). DAPI staining was used to visualize cell death in hAMSCs-treated Panc1 cells. The results showed an up-regulation of TNF-α, IL-1β, IL-8, p-IKK, p-IKKα, p-IKKβ, p-IκB, p53, PUMA, Caspase 3, and NF-κB (p50/p65) as well as a down-regulation of IKβ. These findings suggest that the secretome of hAMSCs promotes both inflammation and apoptosis in Panc1 pancreatic cancer cells simultaneously.

## Highlights

• Pancreatic cancer is a type of cancer with a high mortality rate worldwide.
• Nowadays, stem cell therapy shows promise as a potential treatment option.
• Secretome of stem cells activates TNF-α/NF-kB signaling pathway in Panc1 cells.
• Secretome of stem cells induces expression of IL-8 and IL-1β in Panc1 cells.
• Secretome of stem cells can induce apoptosis in Panc1 cells.

## 1 Introduction

The second most prevalent cause of mortality worldwide among humans is cancer. Pancreatic cancer is considered one of the most deadly malignancies globally. The poor prognosis of pancreatic cancer leads to its rapid spread to the bloodstreams and other distant organs [1,2]. Current strategies for treating pancreatic cancer, such as chemotherapy and radiotherapy, are not effective. Therefore, there is a need to discover new specific targets and therapeutic platforms for pancreatic cancer. Recently, mesenchymal stem cells (MSCs) have been investigated for their potential in pancreatic cancer therapy. MSCs are extensively studied due to their ability to combat tumors, reduce inflammation and target tissues. The microenvironment of MSCs contains various growth factors, cytokines, chemokines, extracellular vesicles (EVs), and microRNA that help to regulate cellular signaling pathways and inflammatory responses in the host tissue [[3], [4], [5], [6], [7], [8], [9], [10]].

Moreover, it has been shown that tumor necrosis factor (TNF)-α is a critical cytokine that mediates cancer-related inflammation and promotion of cancer [11,12]. Nuclear factor-κB (NF-κB) is a transcription factor family that can be activated by TNF-α. Upon activation of NF-κB, the inhibitor of κB (IκB) kinase (or IKK) complex is also activated. The activated IKK complex, which contains IKKα, IKKβ (the catalytic subunits), and IKKγ (the regulatory subunit), is responsible for phosphorylating IκB, leading to its degradation by proteasomes. IKK complex activation depends on Ser177 and Ser181 phosphorylation in the activation loop of IKKβ and in IKKα (Ser176 and Ser180). As a result, free NF-κB translocates to the nucleus (from the cytoplasm), where it attaches to DNA to regulate subsequent gene transcription including TNF-α, IL-8, and IL-1β [[13], [14], [15]]. Furthermore, NF-κB activity has been involved in promoting tumor initiation and development in some cancer cells by stimulating cell proliferation and inhibiting apoptosis [[16], [17], [18]]. However, there have also been studies showing a tumor-suppressive role of NF-κB [19]. Therefore, additional studies are needed to completely understand the complex role of NF-κB in cancer progression.

As a transcription factor, p53 regulates various cellular signaling pathways, including apoptosis, by elevating the expression of pro-apoptotic genes such as PUMA. It acts as a tumor suppressor, with p53 mutations found in 50 % of human cancers [20].

In the current study, we evaluated the effects of the secretome of human mesenchymal stem cells (hAMSCs) on Panc1 pancreatic cancer cells through the TNF-α/NF-κB signaling pathways. We utilized a co-culture system utilizing 6-well Transwell plates. Following a 72-h period, we analyzed cell death in Panc1 cells after treatment with hAMSCs through the TNF-α/NF-κB signaling pathways. Our results showed an up-regulation of TNF-α, IL-1β, IL-8, NF-κB (p50/p65), p-IKK, p-IKKα, p-IKKβ, p53, PUMA, and p-IκB, with a down-regulation of IκB. These results suggest an increase in inflammation in hAMSCs-treated Panc1 cells.

## 2 Materials and methods

### 2.1 Cell culture

HAMSCs (Cat No: C10680, human, female, newborn, spindle-shaped) were obtained from the Iranian Biological Resource Center located in Tehran, Iran, while Panc1 pancreatic cancer cells (NCBI No: C556) were acquired from the Pasteur Institute, also situated in Tehran, Iran. The cells were characterized as MSCs, exhibiting negativity for CD33 and CD45, and positivity for CD90, CD73, and CD105. The basal culture media was DMEM enriched with 10 % FBS, 100 μg/mL penicillin G/streptomycin, and 1 % l-glutamine. All cell conditions followed the guidelines outlined in our previous report [7].

### 2.2 The co-culture system (MSCs and Panc1)

Panc1 cancer cells (15 × 10^4^) were initially seeded in a six-well plate (on the lower side) with a pore diameter of 0.4 μm. The following day, MSCs were seeded at the same density (MSCs: Panc1 of 1:1) on the upper side of a polycarbonate transmembrane filter in a Transwell filter system (BD Falcon, Bedford, MA, USA) as described in our previous study. After a duration of 72 h, Panc1 cells (both control and treated) were used for ELISA assay and Western blot analysis [7].

### 2.3 Western blot

Anti-p-IKKα (T23; sc-101706), anti-B-actin (C4: sc-47778), anti-IκBα (H-4; sc-1643), anti-IKKα (B-8: sc-7606), anti-IL-1β (E7-2-hlL1β; sc:32294), anti-IKKβ (H-4; sc-8014), anti-IL-8 (C-11: sc-376750), and anti-p53 (DO-1; sc-126) were purchased from Santa Cruz Biotechnology. Anti-TNFα (ab6671), anti-PUMA (ab9643), anti- NF-κB p65 (ab16502), and anti-p-IκBα (S32; ab133462) were provided by Abcam. Anti-p-IKKβ (T199; PA5-38280), anti-Caspase 3 (D3R6Y), and anti-p-IKKα/β (S176/180; 16A6) were purchased from Invitrogen and Cell Signaling Technology respectively. All antibodies were served as primary antibodies for immunoblotting and the conditions were consistent with our earlier findings [7]. After a period of 72 h, Panc1 cells were collected and lysed in a buffer composed of 50 mM Tris–HCl (pH 7.5), 150 mM NaCl, 1 mM EDTA, 1 % Triton X-100, 2 mM Na3VO4, and 1 mM PMSF. The total cell lysates (TCLs) were subjected to sodium dodecyl sulfate-polyacrylamide gel electrophoresis (SDS-PAGE). Proteins were transferred to polyvinylidene difluoride (PVDF) membrane filters (Millipore), which were then soaked in solutions containing primary antibodies followed by secondary antibodies. The bands were visualized using Western blot chemiluminescence reagent (PerkinElmer Life Sciences), and the intensity of the detected bands was evaluated using ImageJ software. For the stripping process, the membrane was immersed in the stripping solution to (containing mercaptanol, Tris, and SDS) at 37 °C for 3 min, then washed with TBST prior to re-blocking and re-probing the membrane.

### 2.4 Cellular viability assay

The impact of hAMSCs secretome on the viability of Panc1 cancer cells was investigated by 3-(4, 5-dimethylthiazol-2-yl)-2, 5-diphenyl tetrazolium bromide (MTT) assay (MTT assay kit, Bio IDEA, Cato: BI1017, Iran), which was previously described [7].

### 2.5 DAPI staining

The DAPI (4, 6-Diamidino-2-phenylindole dihydrochloride) staining assay was utilized to determine morphological changes in nucleus of hAMSCs-treated Panc1 cancer cells after 24 h. In summary, Panc1 cancer cells were cultured in 6-well plates (5 × 10^4^ cells/well) with 12 mm cover-slips. They were then treated with hAMSCs secretome for a duration of 24 h. The cells were then fixed with 3.7 % paraformaldehyde, permeabilized with 0.5 % (w/v) Triton X-100, 1 % BSA (w/v) for 5 min, followed by washing in PBS, and stained with DAPI (Sigma-Aldrich, USA). All images were obtained using an inverted fluorescent microscope (Nikon Eclipse Ti-E) [7].

### 2.6 Enzyme-linked immunosorbent assay (ELISA)

Panc1 cancer cells were treated with hAMSCs secretome for 48 h. The levels of IL-1β, IL-8, TNFα, and NF-κB p65 in the supernatant were measured using ELISA kits (R&D Systems, Minneapolis, MN, USA) following the manufacturer's instructions. Standard and sample wells were arranged as instructed. The plates were sealed with a film, then incubated at 37 °C for 30 min before discarding the liquid and washing the plates five times. Enzyme-labeled reagent (50 μl) was added to each well, and incubated at 37 °C for 30 min, excluding the blank wells. After washing, A and B chromogens (50 μl) were added and incubated in the dark at 37 °C for 15 min. Then, the reaction was stopped by adding 50 μl of stop solution. The blank well was set to 0, and the absorbance of each well was calculated using a microplate reader (Biorad, iMark) within 15 min for an OD value at 450 nm.

### 2.7 Statistical analysis of the data

The data was analyzed and graphed using SPSS 22 (Chicago, IL, USA) and GraphPad Prism 7 software, following the methods outlined in our previous study. The data were presented as means ± standard deviation (SD). Experiments were conducted in triplicate. Comparisons between groups were performed using an independent sample *t*-test. A p-value of less than 0.05 was considered to be statistically significant [7].

## 3 Results

### 3.1 Induction of TNF-α in hAMSCs secretome-treated Panc1 cells

**Fig. 1:**
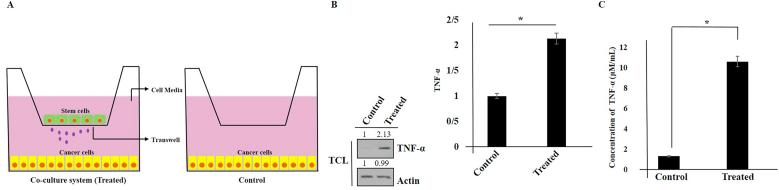
Schematic model of the co-culture system used in this study (A). The induction of TNF-α expression in hAMSCs secretome-treated Panc1 cells using Western blot and an ELISA kit (B and C). Actin was used as an internal control (TCL: total cell lysate). Data represent mean ± SD of three independent experiments. ∗p < 0.05 was considered statistically significant.

In pancreatic cancer cell lines and pancreatic tumor samples, high expression of TNF-α has been previously reported [21]. In our experiments, we cultured Panc1 cancer cells and hAMSCs cells using a Transwell co-culture system (Fig. 1A). After 72 h, we then measured the expression of TNF-α using Western blotting and ELISA kits (Fig. 1B and C). Our results indicated an increase in TNF-α levels in Panc1 cells after treatment with hAMSCs secretome.

### 3.2 Elevation of NF-κB p65/p50 and suppression of IκB in hAMSCs secretome-treated Panc1 cells

**Fig. 2:**
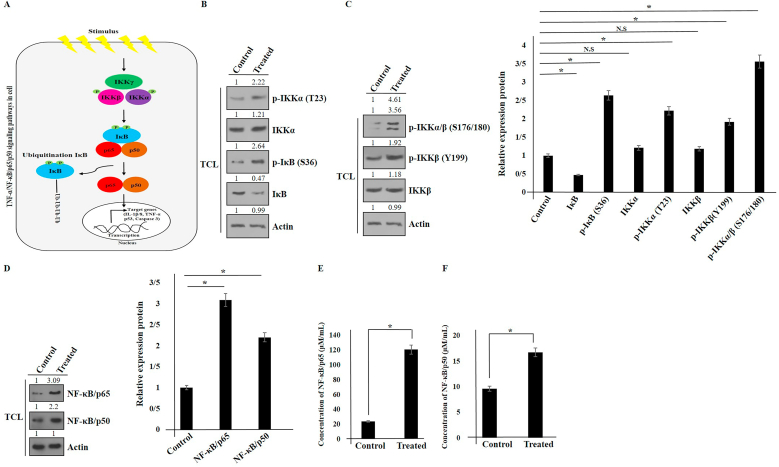
Schematic model of NF-κB activation (A). The expression of p-IKK, p-IKKα/β, NF-κB (p50/p65), IκB, and p-IκB proteins was analyzed using Western blot in hAMSCs-treated Panc1 cancer cells (B, C, and D). Actin was used as an internal control (TCL: total cell lysate). The induction of NF-κB (p50/p65) expression in hAMSCs secretome-treated Panc1 cells was measured using an ELISA kit (E and F). Data represent mean ± SD of three independent experiments. ∗p < 0.05 was considered statistically significant. N.S: No significant.

TNF-α enables to activate NF-κB, a transcription factor family. Upon activation of NF-κB, the IKK complex (containing IKKγ, IKKα, and IKKβ) is activated. Activated IKK phosphorylates IκB, leading to its degradation by proteasomes. Subsequently, free NF-κB translocates from cytoplasm to the nucleus and attaches to DNA, regulating downstream gene transcription such as TNF-α, IL-8, and IL-1β [[13], [14], [15]] (Fig. 2A). In the classic NF-κB pathway, both IKKα and IKKβ phosphorylation, especially the phosphorylation of serines on IKKβ, are required for NF-κB activation. In this study, a co-culture system was utilized to detect NF-κB activation and upstream proteins in the NF-κB pathway. Phosphorylation of IKKα/β and inhibition of IκB were observed (see Fig. 2B-D). Additionally, high expression of NF-κB p65/p50 in hAMSCs secretome-treated Panc1 cells was demonstrated through Western blot and ELISA methods.

### 3.3 IL-1β and IL-8 elevation in hAMSCs secretome-treated Panc1 cells

**Fig. 3:**
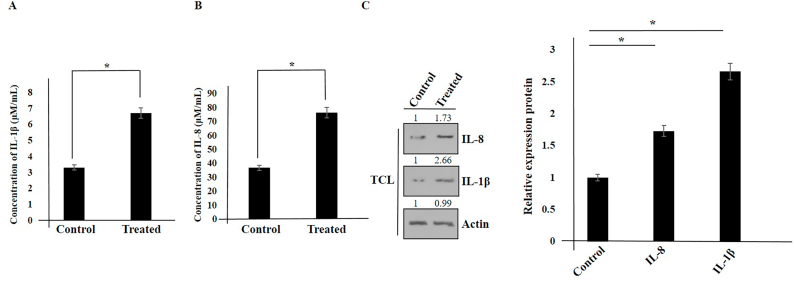
The induction of IL-1β and IL-8 expression in hAMSCs secretome-treated Panc1 cells was analyzed using an ELISA kit and Western blot (A–C). Data represent mean ± SD of three independent experiments. ∗p < 0.05 was considered statistically significant. Actin was used as an internal control (TCL: total cell lysate).

IL-1β and IL-8 are induced by activated NF-κB. Therefore, we analyzed the expression of IL-8 and IL-1β in hAMSCs secretome-treated Panc1 cells. Panc1 cancer cells were initially seeded in a co-culture system using 6-well Transwell plates. The following day, hAMSCs were seeded in a Transwell filter system (at the same density on the upper surface of a polycarbonate transmembrane filter). After 72 h, Panc1 cells (treated and control) were subjected to Western blot and ELISA methods. Our results indicated that after treatment with hAMSCs secretome, the induction of IL-8 and IL-1β was observed in Panc1 cells (Fig. 3A-C).

### 3.4 Apoptosis induction through activation of Caspase3, p53, and PUMA in hAMSCs secretome-treated Panc1 cells

**Fig. 4:**
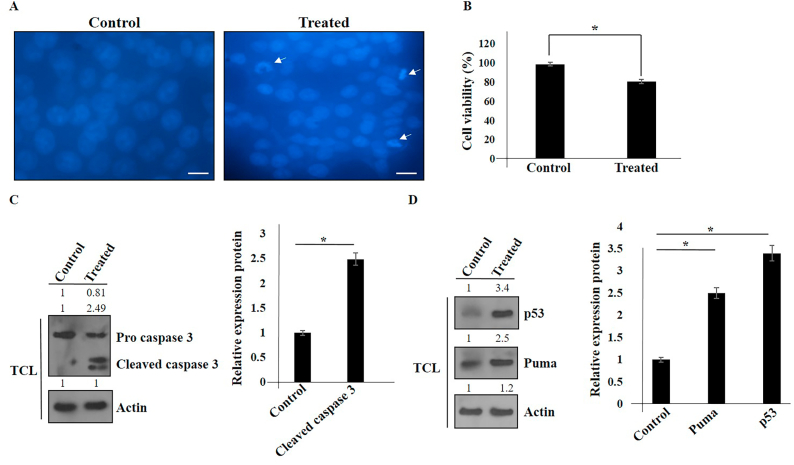
Cellular apoptosis induction in hAMSCs-treated Panc1 cancer cells using DAPI. The experiments were conducted three times (original microscope magnification, 40X, Scale bar, 10 μm) (A). Inhibitory effect of hAMSCs on Panc1 cancer cells using MTT assay. Data represent mean ± SD of three independent experiments. ∗p < 0.05 was considered statistically significant (B). The expression of p53, PUMA, and Caspase 3 proteins was shown in hAMSCs-treated Panc1 cells using Western blot (C, D). Actin was used as an internal control (TCL: total cell lysate).

NF-κB plays a critical role in the escape from apoptosis in some cancer cells leading drug resistance of tumor cells [[16], [17], [18]]. Therefore, we evaluated the impacts of the secretome of hAMSCs on Panc1 cancer cells. In this study, Panc1 cancer cells were seeded in 6-well plates and subsequently treated with hAMSCs secretome for 24 h. The cells were then stained with DAPI (Fig. 4A and B). Additionally, Panc1 cancer cells were first seeded in a co-culture system using 6-well Transwell plates. The following day, hAMSCs were seeded in a Transwell filter system. After 72 h, Panc1 cells (treated and control) were subjected to Western blot analysis to assess the related proteins (Fig. 4C and D). Our results showed that the promotion of apoptosis through the activation of p53, PUMA, and Caspase 3 was observed.

## 4 Discussion

**Fig. 5:**
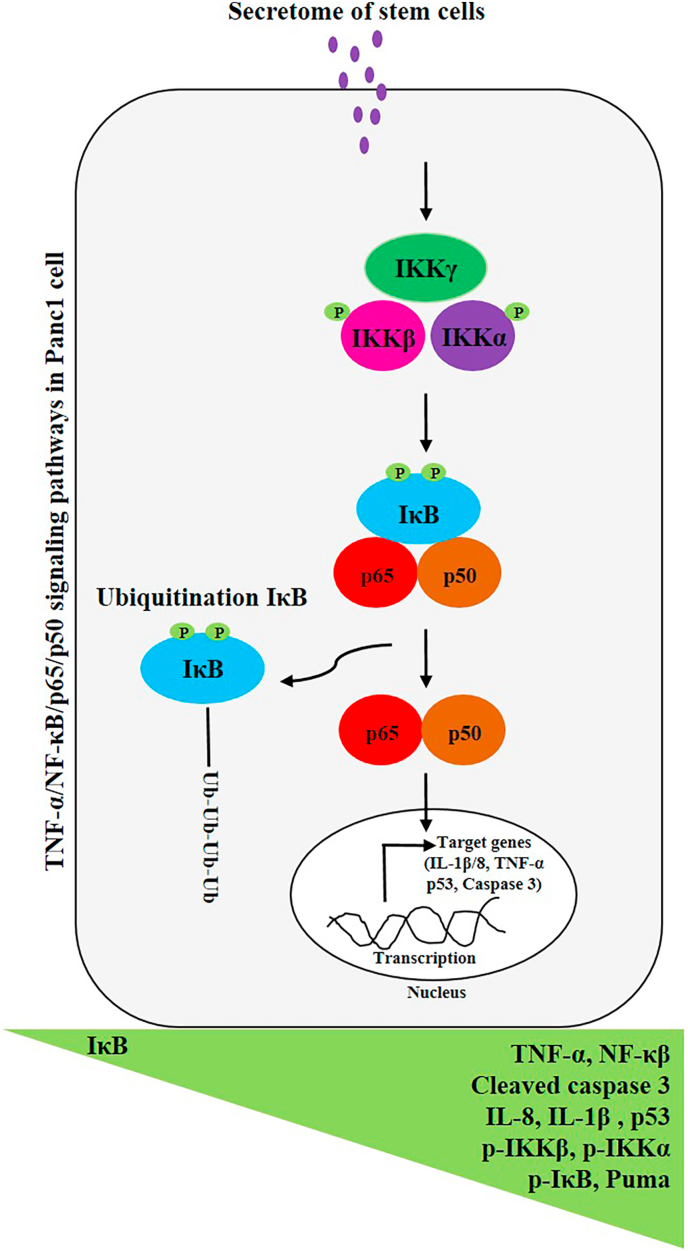
A schematic summary of the present study. HAMSCs secretome enables the induction of TNF-α, IL-1β, IL-8, p-IKK, p-IKKα, p-IKKβ, p-IκB, p53, PUMA, Caspase 3, NF-κB (p50/p65) and down-regulation of IKβ expressions in Panc1 cancer cells.

Pancreatic cancer is one of the most aggressive tumors, associated with high mortality rates and a poor prognosis globally. Due to pancreatic cancer insensitivity to current treatment options (such as radiotherapy, chemotherapy, and immunotherapy), mainly resulting from apoptosis resistance, the survival rate of pancreatic cancer is low [22,23]. In the current study, we aim to assess the effects of hAMSCs secretome on Panc1 cancer cells via TNF-α/NF-κB signaling pathways. Our results suggest simultaneous induction of inflammation and apoptosis by the secretome in hAMSCs-treated Panc1 pancreatic cancer cells. We showed up-regulation of TNF-α, IL-1β, IL-8, p-IKK, p-IKKα, p-IKKβ, p-IκB, p53, PUMA, cleaved caspase 3, NF-κB (p50/p65) and down-regulation of IκB (see Fig. 5).

TNF-α (as a pro-inflammatory cytokine), can act either as a promoter of cancer or a tumor suppressor. It appears that the balance between TNF-induced survival and death signaling is crucial in determining the biological effects of TNF-α in cells. Previous studies have indicated that TNF-α promotes apoptosis in human malignant keratinocytes, while normal keratinocytes remain unaffected [24]. The main anticancer property of TNF-α is its ability to induce cell death [25]. Additionally, the roles of TNF-α in pancreatic cancer have been documented [26,27].

NF-κB proteins act as transcription factors and are activated in response to inflammatory stimuli. In approximately 70 % of pancreatic cancers, NF-κB activity is persistently active [28]. Interestingly, this continual activation of NF-κB appears to be necessary for resistance to TNF-α-induced apoptosis [29,30]. In response to chemotherapeutic agents, NF-κB has also been reported to suppress apoptosis [31,32]. In the current study, we demonstrated the simultaneous induction of inflammation and apoptosis through the TNF-α/NF-κB signaling pathway by the secretome of hAMSCs in Panc1 pancreatic cancer cells. It is worth noting that our previous results have shown the induction of apoptosis by the secretome of hAMSCs through the up-regulation of Bax and cleaved Caspase 3 and the down-regulation of Bcl2 in various cancer cells [9,10,[33], [34], [35]].

It was shown that IKKα was phosphorylated at T23 by Akt *in vitro,* and the T23A mutant reduced NF-κB activity in BT20 breast cancer cells. Moreover, it was determined that AKT serves as a crucial mediator in the TNFα-induced activation of NF-κB by phosphorylating IKKα at T23 [36]. In this study, we demonstrated that the induction of phosphorylation of IKKα at T23 in Panc1 pancreatic cancer cells by hAMSCs' secretome. Additionally, it was shown that the inducible phosphorylation sites of IκB (S32/36) are crucial for ubiquitination, leading to IκB degradation and NF-κB activation. Importantly, IκB mutants (A32/36) act as critical inhibitors of NF-κB activation [37]. We also observed the induction of phosphorylation of IκB (at S36), supporting NF-κB activation in Panc1 pancreatic cancer cells by hAMSCs' secretome. In fact, NF-κB regulates many pro-and anti-apoptotic genes in both the extrinsic and intrinsic pathways. p53 is another key protein that regulates apoptosis. NF-κB and p53 antagonistically regulate each other's activity. The balance between NF-κB and p53 is important in governing cell growth and cell death. IKKβ is able to phosphorylate p53 to enhance its ubiquitination and degradation (independently of Mdm2) [[38], [39], [40]].

Panc1 is a pancreatic cancer cell line that contains mutant-type p53 (R273H), and Panc1 possesses only one copy of *p53* gene. It has been indicated that mutant p53 loses its ability to bind to the PUMA promoter due to changes in its configuration, which could contribute to the inhibition of apoptosis and may be a factor in chemotherapy resistance [41]. Additionally, it was noted that overexpression of p53 in mutant p53 cells could induce PUMA. Interestingly, we have shown that the secretome of hAMSCs promotes apoptosis by activating of p53, PUMA, and Caspase 3 in Panc1 pancreatic cancer cells treated with hAMSCs. In this regard, it has been shown that natural compounds such as Butein, can restore the wild-type-like conformation and DNA-binding ability of mutp53-R273H. This is achieved by disrupting the interaction between mutp53 and Hsp90 (heat shock protein 90) and increasing the acetylation of Hsp90. This leads to the reestablishment of p53's transcriptional activation of p53 target genes, such as PUMA which induces apoptosis in tumor cells [42]. Further investigation will be required to elucidate the precise mechanism involved.

It was previously reported that IL-8 and IL-1β levels are elevated in pancreatic cancer patients. Moreover, it was suggested that IL-8 and IL-1β are critical markers for the prognosis and drug resistance of pancreatic cancer [43,44]. In the current study, we found that the secretome of hAMSCs did not show inhibitory effects on IL-8 and IL-1β production in Panc1 cancer cells. In this respect, it was found that the Fas/FasL pathway mediates apoptosis and induces IL-8 in bronchiolar epithelial cells *in vitro* [45]. Another study showed that IL-1β and TNF-α cytokines regulate the activation of the apoptotic machinery in human chondrocytes [46]. More experiments will be required to elucidate the exact molecular mechanism.

Nowadays, stem cell transplantation and secretome therapy have emerged as novel approaches in regenerative medicine. In secretome therapy, the risk of immunogenicity, DNA mutation, tumor formation, and vascular obstruction is low. Easy production, storage, evaluation of safety and dosage of secretome are other advantages of secretome therapy. However, the potent advantages of stem cell transplantation lie in their ability to differentiate into specific tissues and migrate to the site of injury [47].

## 5 Conclusions

In summary, pancreatic cancer is a type of cancer that typically presents with no symptoms, has an unfavorable prognosis, and a high global mortality rate. Among the various platforms in cancer therapy, stem cell therapy shows promise as a potential treatment option. Our results suggest that, despite the activation of the TNF-α/NF-κB signaling pathway and high expression of IL-8 and IL-1β, the secretome of hAMSCs can promote apoptosis in Panc1 pancreatic cancer cells through the upregulation of p53, PUMA, and Caspase3. Nonetheless, additional experiments are required to fully elucidate the underlying molecular mechanisms.

## Funding

None.

## CRediT authorship contribution statement

**Aynaz Khalafi:** Formal analysis. **Fatemeh Safari:** Conceptualization, Formal analysis, Investigation, Supervision, Validation, Visualization, Writing – original draft, Writing – review & editing.

## Declaration of competing interest

The authors declare no competing financial interests.

## Data Availability

Data will be made available on request.
